# Pathotypic characterization of Newcastle disease virus isolated from vaccinated chicken in West Java, Indonesia

**DOI:** 10.14202/vetworld.2017.438-444

**Published:** 2017-04-22

**Authors:** Dwi Desmiyeni Putri, Ekowati Handharyani, Retno Damajanti Soejoedono, Agus Setiyono, Ni Luh Putu Ika Mayasari, Okti Nadia Poetri

**Affiliations:** 1 Study Program of Animal Biomedical Science, IPB Graduate School, Bogor Agricultural University, Bogor, Indonesia; 2 Study Program of Animal Husbandry, Department of Animal Husbandry, State Polytechnic of Lampung, Lampung, Indonesia; 3 Department of Veterinary Clinic Reproduction and Pathology, Faculty of Veterinary Medicine, Bogor Agricultural University, Bogor, Indonesia; 4 Department of Animal Diseases and Veterinary Public Health, Faculty of Veterinary Medicine, Bogor Agricultural University, Bogor, Indonesia

**Keywords:** F gene, M gene, molecular pathotyping, Newcastle disease virus, vaccinated chickens, virulence

## Abstract

**Aim:**

This research was conducted to differentiate and characterize eight Newcastle disease virus (NDV) isolates collected from vaccinated chicken at commercial flocks in West Java, Indonesia, in 2011, 2014 and 2015 by pathotype specific primers.

**Materials and Methods:**

A total of eight NDV isolates collected from clinical outbreaks among commercial vaccinated flocks in West Java, Indonesia, in 2011, 2014, and 2015 were used in this study. Reverse transcription-polymerase chain reaction was used to detect and differentiate virulence of NDV strains, using three sets of primers targeting their M and F gene. First primers were universal primers to detect NDV targeting matrix (M) gene. Other two sets of primers were specific for the fusion (F) gene cleavage site sequence of virulent and avirulent NDV strains.

**Results:**

Our results showed that three isolates belong to NDV virulent strains, and other five isolates belong to NDV avirulent strains. The nucleotide sequence of the F protein cleavage site showed ^112^K/R-R-Q/R-K-R/G-F^117^ on NDV virulent strains and ^112^G-K/R-Q-G-R-L^117^ on NDV avirulent strain.

**Conclusion:**

Result from the current study suggested that NDV virulent strain were circulating among vaccinated chickens in West Java, Indonesia; this might possess a risk of causing ND outbreaks and causing economic losses within the poultry industry.

## Introduction

Newcastle disease (ND) is a highly contagious and fatal disease of poultry [1], due to the potential for devasting losses [2] and categorized in list A disease by the Office International des Epizooties (OIE) [3-5]. The diseases were caused by ND virus (NDV) or Avian Paramyxovirus Type 1, which belongs to the genus Avulavirus in the family of Paramyxoviridae [6]. NDV infection would lead to a broad range of clinical signs, such as asymptomatic enteric to systemic infection with up to 100% mortality [7]. Based on the severity of the disease in chickens, NDV is categorized into asymptomatic enteric (avirulence), lentogenic (low virulence), mesogenic (intermediate virulence), and velogenic (high virulence) pathotypes [6]. The velogenic and mesogenic (virulent) strains have been identified as the causative agent of ND outbreaks in many countries. The avirulent strains have been used as live vaccines to control the disease; however, ND outbreaks have been reported in vaccinated poultry consistenly [4].

In general, NDV characterization was determined using virus isolation followed by *in vivo* tests such as intracerebral pathogenicity index (ICPI), intravenous pathogenicity index (IVPI), and mean death time (MDT) in specified pathogen free (SPF) chicken embryo/birds for pathotyping [3,8]. However, molecular methods - such as reverse transcription-polymerase chain reaction (RT-PCR) and amino acid sequencing - were developed for NDV detection and pathotyping [4,9,10]. The previous study has improved RT-PCR using pathotype specific primers to detect and differentiate virulence of NDV strains; this technique seems to be more efficient for NDV pathotyping [11,12]. The pathotype specific primers set was conducted based on nucleotides sequence at the F gene cleavage site, which has been shown to be a major determinant of NDV virulence [13-15].

Indonesia has a large poultry population, and West Java is the province with the largest poultry population i.e. 497,814,132 or 44.64% from national poultry population [16]. West Java has experienced with recurrent of NDV outbreak among vaccinated commercial flocks [17]. The previous study reported that NDV genotype VII virulent strain was causing the outbreaks among backyards and commercials flock since 2009 [17,18]. NDV genotype VII was also isolated from live bird market in some district in West Java, such as Bogor, Sukabumi, and Tangerang [19]. Understanding the pathotypic character of NDV isolated from clinical outbreaks were important to control the diseases in West Java; however, such information is limited. Therefore, this study was designed to differentiate and characterize NDV isolates collected from clinical outbreaks among commercial vaccinated flocks in West Java, Indonesia, in 2011, 2014, and 2015 by pathotype specific primers. The virulence of NDVs was evaluated by molecular analyses of the nucleotide and deduced amino acid sequences of the F gene. Our results might provide a better understanding of NDV circulating in the region and contribute for controlling the diseases.

## Materials and Methods

### Ethical approval

This study were performed according to the regulations for Research in Animal Health of Indonesian Law on Livestock and Animal Health (UU/18/2009, article 80).

### Virus

#### NDV isolates

A total of eight NDV isolates collected from clinical outbreaks among commercial vaccinated flocks in West Java, Indonesia, in 2011, 2014, and 2015 were used in this study. Origin and year of isolation of NDV isolates were shown in Table-1. These isolates are NDV/Ck/BGR/11, NDV/Ck/GS/14, NDV/Ck//JP/14, NDV/Ck/LG/15, NDV/Ck//CJR/15, NDV/Ck/BGR/15, NDV/Ck/TRG/15, and NDV/Ck/LWG/15. Two NDV strains representing different pathotypes: NDV/Lasota (avirulent) and NDV/Sato (virulent) were used as representative strains. Before testing in RT-PCR, all the samples were propagated once into SPF embryonated chicken eggs (ECEs) and allantoic fluid were used for further studies.

**Table-1:** Data and characteristics of the NDV isolates used for the investigation.

Isolate^a^	Origin^b^	Year of isolation	RT-PCR result^c^			Cleavage site sequence	Pathotype

			M	FA/FB	FA/FC		
NDV/Ck/BGR/11	Bogor	2011	+	+	-	R-R-Q-K-R-F	Virulent
NDV/Ck/GS/14	Gunung Sindur	2014	+	+	-	K-R-R-K-R-F	Virulent
NDV/Ck/JP/14	Jampang	2014	+	-	+	G-K-Q-G-R-L	Avirulent
NDV/Ck/LG/15	Legok	2015	+	-	+	G-K-Q-G-R-L	Avirulent
NDV/Ck/CJR/15	Cianjur	2015	+	-	+	G-R-Q-G-R-L	Avirulent
NDV/Ck/BGR/15	Bogor	2015	+	-	+	G-R-Q-G-R-L	Avirulent
NDV/Ck/TRG/15	Tangerang	2015	+	-	+	G-K-Q-G-R-L	Avirulent
NDV/Ck/LWG/15	Leuwiliang	2015	+	+	-	R-R-Q-K-G-F	Virulent

a All isolates were collected from vaccinated chickens showing clinical sign of ND,

b All origin were located at West Java Province,

c M=Matriks gene (universal primer for detecting NDV); FA/FB=F gene (virulent NDV); FA/FC=F gene (avirulent NDV), Lanes 9–10 are Sato and 11–12 are Lasota (used as positive control). NDV=Newcastle disease virus, RT-PCR=Reverse transcription-polymerase chain reaction

#### RNA isolation

RNAs of the viruses were extracted from allantoic fluids using QIAamp^@^ Viral RNA Mini Kit catalog number 52904 (Qiagen, Germany) according to manufacturer instruction [20]. 140 µl of sample suspension was used for extraction, and RNA was diluted in a final volume 60 µl and store at −80°C.

### RT-PCR

#### Amplification

RT-PCR was performed using one-step RT-PCR kit (Qiagen, Germany) according to manufacturer instruction. RT-PCR reaction mixture of each sample consisted of 2 µl of dNTPs mix (10 mM), 2 µl of forward primer (10 pM), 2 µl of reverse primer (10 pM), 2 µl of purified template RNA, 10 µl of 5× Onestep RT-PCR Qiagen buffer, 30 µl of RNase-free water, and 2 µl one-step RT-PCR enzyme mixed in a final volume of 50 µl. Amplification for M gene was setup as 45°C for 60 min followed by initial denaturation at 95°C for 5 min and 35 cycles of denaturation at 95°C for 30 s, annealing at 50°C for 30 s, extension at 72°C for 40 s and final extension at 72°C for 10 min. Amplification for F gene were performed according to the following protocol: 45°C for 60 min for c-DNA synthesis followed by initial denaturation at 94°C for 5 min, and 35 cycles of 94°C for 1 min, 50°C for 1 min, 72°C for 1 min, with a final elongation step of 5 min for 72°C.

#### Primers

Three sets of primers were used for amplification. First primers set are NDV-MF/MR were universal primers targeting for M gene. Another two set of primers targeting F gene cleavage site: NDV-FA/FB were specific for virulent NDV and NDV-FA/FC were specific for avirulent NDV strains [11]. Nucleotide sequences of all primers were presented in Table-2.

**Table-2:** Matrix (M) and Fusion (F) primers sequence used for the investigation.

Gene	Code	Sequence	Position (bp)
Matrix	MF	5′-TCGAGTCTGTACAATCTTGC-3′	232
Matrix	MR	5′-GTCCGAGCACATCACTGAGC-3′	
Fusion	FA	5′-TTGATGGCAGGCCTCTTGC-3′	141–159
Fusion	FB	5′-AGCGT (C/T) TCTGTCTCCT-3′	395–380
Fusion	FC	5′-G (A/G) CG (A/T) CCCTGT (C/T) TCCC-3′	395–380

#### Electrophoresis

PCR products were separated in 1.5% agarose gel in 1× Tris-acetate-ethylenediaminetetraacetic acid buffer stained with ethidium bromide, compared with molecular mass marker and visualized by ultraviolet transillumination.

### Nucleotide sequencing

#### Sequencing of PCR product

The positive results of PCR products were sequenced using BigDye^®^ Terminator v3.1 cycle Sequencing Kit (Thermo Fisher Scientific, USA) according to manufacturer instruction. The first stage began with the purification of PCR products using Centricon^®^-100 columns (Millipore, USA). Purified PCR products were sequenced by First Base Company (Malaysia) with the primer NDV-FA. The nucleotide sequencing was performed according to the following protocol: denaturation at 96°C for 1 min, followed by 25 cycles of 96°C for 10 s, 50°C for 5 s, and 60°C for 4 min, with a final elongation step of 5 min for 72°C. The final stage was the purification of the product cycle sequencing using Centri-Sep™ spin columns (Thermo Fisher Scientific, USA) according to manufacturer instruction.

#### Analysis of nucleotide sequence

The obtained sequence was edited using BioEdit Sequence Alignment Editor Version 7.0.9.0. Alignment of the sequences was performed using MEGA version 6 [21].

## Results

### M and F gene RT-PCR

**Figure-1:**
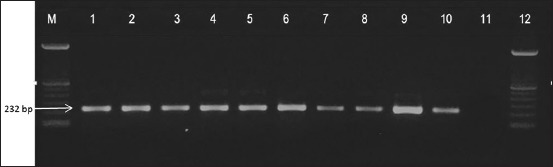
Matrix gene amplification results. Polymerase chain reaction product size of 232 bp. The amplicons were electrophoresed in 1,5% agarose gel. Lanes: M - Molecular size marker; Lanes 1–8 are Newcastle disease virus (NDV) field isolates, Lanes 9 NDV/Sato and 10 are NDV/Lasota (used as positive control); Lane 11 is non template control.

**Figure-2:**
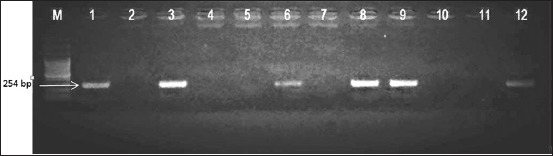
Fusion gene amplification results. Reverse transcription-polymerase chain reaction amplification of the Newcastle disease virus fusion gene using FA (forward) and FB, FC (reverse) primer combination, which gave a product size of 254 bp. The amplicons were electrophoresed in 1.5% agarose gel. Lanes: M - Molecular size marker; Lanes 1, 3, 5, 7, 9, 11 (FA/FB), lanes 2, 4, 6, 8, 10, 12 (FA/FC), Lane 1–2 - Newcastle disease virus (NDV)/Ck/BGR/11, 3–4 - NDV/Ck/GS/14, 5–6 - NDV/Ck/JP/14, 7–8 - NDV/Ck/LG/15, Lanes 9–10 are Sato and 11–12 are Lasota (used as positive control).

**Figure-3:**
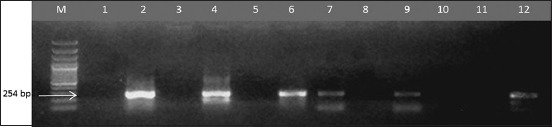
Reverse transcription-polymerase chain reaction amplification of the Newcastle disease virus fusion gene using FA (forward) and FB, FC 2 (reverse) primer combination, which gave a product size of 254 bp. The amplicons were electrophoresed in 1.5% agarose gel. Lanes: M- molecular size marker; Lanes 1, 3, 5, 7, 9, 11 (FA/FB), Lanes 2, 4, 6, 8, 10, 12 (FA/FC), Lane 1–2- Newcastle disease virus (NDV)/Ck/CJR/15, 3–4- NDV/Ck/BGR/15, 5-6- NDV/Ck/TRG/15, 7–8 NDV/Ck/LWG/15. Lanes 9–10 are Sato and 11–12 are Lasota (used as positive control).

M gene amplification results are presented in Figure-1 and Table-1. All isolates showed specific band at 232 bp indicating that all isolates were NDV. Identification of NDV virulent and avirulent strain was determined using combination of F gene primer (pathotype specific primers) developed by Kant *et al*. [11]. F gene amplification results are presented in Figures-2 and 3; Table-1. Three isolates could be amplified using NDV-FA/FB primer, while other five isolates could be amplified using NDV-FA/FC primer. Based on F gene amplification result, three isolates which is NDV/Ck/BGR/11, NDV/Ck/GS/14, and NDV/Ck/LWG/15 were belong to virulent NDV strains, while other five isolates: NDV/Ck/JP/14, NDV/Ck/LG/15, NDV/Ck//CJR/15, NDV/Ck/BGR/15, and NDV/Ck/TRG/15 were belong to avirulent NDV strains.

### Cleavage site sequencing

**Figure-4:**
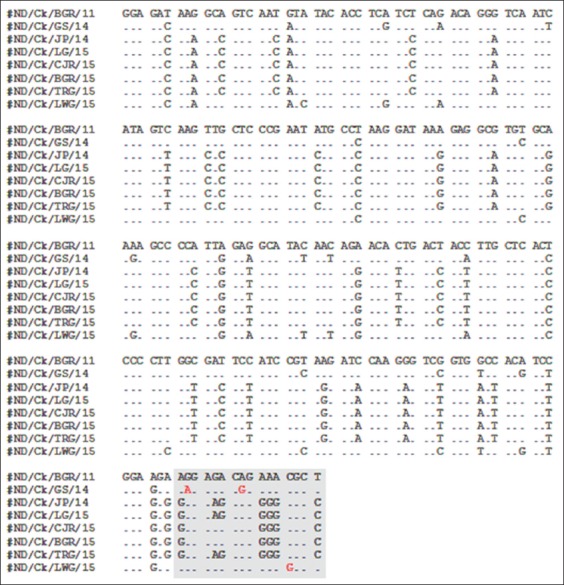
Partial nucleotide sequences of Newcastle disease isolates. Currently, part of F gene from position 136 to 349 is shown. Estimation of cleavage sites were marked with grey. Different nucleotide of cleavage sites among virulent isolates were marked in red font.

Nucleotide sequence of F gene cleavage site is presented in Figure-4. NDV virulent strains have multiple basic amino acid arginine (R) or lysine (K) at the fusion (F) cleavage site at residues 112-113 and 115-116 and showed motif ^112^K/R-R-Q/R-K-R/G-F^117^. NDV/Ck/BGR/11 showed motif R-R-G-K-R-F, NDV/Ck/GS/14 showed motif K-R-R-K-R-F, and NDV/Ck/LWG/15 showed motif R-R-Q-K-G-F on its F gene cleavage site sequence. NDV/Ck/LWG/15 has different pattern with other two virulent isolates; however, the sequence homology with NDV/Ck/GS/14 were 96% (with the nucleotide in position 335, 341, and 346 were different). NDV avirulent strains shown motif ^112^G-K/R-Q-G-R-L^117^ on its cleavage site sequence. NDV/Ck/JP/14, NDV/Ck/LG/15, and NDV/Ck/TGR/15 have motif G-K-Q-G-R-L and the NDV/Ck/JP/14 and NDV/Ck/LG/14 shown motif G-R-Q-G-R-L. Cleavage site nucleotide sequence results were in accordance with amplification F gene using pathotype specific primer results (Figure-4).

## Discussion

Indonesia has been an endemic country for ND, despite control strategies that have been done to eradicate the viruses. In general, ND vaccination program has been applied among commercial poultry flocks in Indonesia. However, in 2009 and 2010, clinical ND outbreaks among vaccinated commercial flocks were reported, causing up to 70–80% mortality [18]. Diseases control program need better understanding of circulating viruses characteristic, and this could be achieved by routine surveillance. Diagnosis of diseases among poultry flocks should be performed quickly and accurate.

According to OIE [3], the gold standard methods NDV laboratory diagnosis are virus isolation using ECE aged 9–11 days. NDV pathogenicity was assessed by determining the MDT of embryos, ICPI, and IVPI [4]. However, this method requires a relatively long time for NDV diagnosis, molecular technique such RT-PCR offered faster result [4,22,23].

RT-PCR for NDV detection was first described by Jestin and Jestin [22], and this technique has been modified by Seal *et al*. [24], Aldous and Alexander [4], Rabalski *et al*. [25] using universal primers to detect all NDVs; Kant *et al*. [11], Lai *et al*. [26], and Al-Shammari *et al*. [27] developed pathotype specific primers that enable rapid differentiation of the pathotype; also Kho *et al*. [23] developed nested PCR for detecting NDV. Thus, in our study, we applied RT-PCR method using NDV universal primers [24] and pathotype specific primers [11]. Our result revealed that universal primers (M gene) developed by Seal *et al*. 1995 [24] were able to detect NDV viruses.

Pathotype specific primers developed by Kant *et al*. [11] were able to differentiate NDV virulence. The primers are targeting F gene cleavage site which has the same target sequence of forward primer (FA) and have different sequence of reverse primers (FB and FC). NDV/Ck/BGR/11, NDV/Ck/GS/14, and NDV/Ck/LWG/15 isolates were amplified using NDV-FA/FB primer, and other five isolates NDV/Ck//JP/14, NDV/Ck/LG/15, NDV/Ck//CJR/15, NDV/Ck/BGR/15, and NDV/Ck/TRG/15 were amplified using NDV-FA/FC primer. Our result showed that both NDV virulent and avirulent strain were able to isolated from vaccinated flocks.

Nucleotide sequence of F gene cleavage site was used to predict pathotype of NDV [28]. A molecular basis of pathogenicity has been well established through sequence analysis of F-protein cleavage site. In general, nucleotide sequence at cleavage site of NDV virulent strains at least have three basic amino-acids (multibasic cleavage site) arginine (R) or lysine (K) in positions 112–116 and amino acid phenylalanine (F) at position 117, and NDV avirulent strain have less than three basic amino acids (monobasic cleavage site) in positions 112–116 and amino-acid leucine (L) at position 117 [3,14,28-30]. NDV/Ck/BGR/11 showed motif R-R-G-K-R-F which has the same motif on its F gene cleavage site with earlier NDV isolated in 2009-2012 in Indonesia [19]. However, NDV/Ck/LWG/15 showed motif R-R-Q-K-G-F which is different from other two virulent NDV isolates. This isolat has 96% homology with NDV/Ck/GS/14, however the nucleotide in position 335, 341, and 346 was different. This difference could explained based on alteration of amino acid motif. The alteration of amino acid caused by mutation or substitution was associated with the many diverse genotypes of the virus [31]. NDV/Ck/JP/14, NDV/Ck/LG/15, and NDV/Ck/TGR/15 showed motif G-K-Q-G-R-L and the NDV/Ck/JP/14 and NDV/Ck/LG/14 showed motif G-R-Q-G-R-L. Genotype I and II strains carried the fusion cleavage site motifs ^112^G-K-Q-G-R-L^117^ and ^112^G-R-Q-G-R-L^117^ [31]. The isolates were clustered in genotype I and were identical to the vaccine strain Queensland V4 and other isolates were clustered in genotype II and were very similar to the vaccine strain B1, suggesting that all genotype I and II strains were related to live vaccine strains [31]. Studies comparing the deduced amino acid sequence of the cleavage site of NDV varying in virulence for chickens shown motif ^112^K/R-R-Q/R-K-R/G-F,^117^ whereas avirulent strain of NDV show motif ^112^G-K/R-Q-G-R-L^117^.

Nucleotide sequence of F gene cleavage site results was consistent with the result of F gene amplification using pathotype specific primers as presented in Figure-4, indicating that RT-PCR using pathotype specific primers NDV-FA/FB and NDV-FA/FC were able to differentiated NDV pathotype. The previous study by Wang *et al*. [32] and Ahmadi *et al*. [12] were also able to differentiate NDV pathotype using such primers. However, another study by Tiwari *et al*. [33] showed different phenomenon whereas NDV virulent strain were amplified using both pathotype specific primers NDV-FA/FB and NDV-FA/FC.

Development of molecular techniques such as RT-PCR offers significant advantages on laboratory diagnostic [34]. PCR technique has high sensitivity and specificity for identify pathogen, and were able to identify pathogen within species. Using this technique, we were able to do nucleotide sequence which notify difference or subtitution or mutation on species amino acid sequence. This technique were often used on molecular epidemiology study [35]. Our current study showed that NDV avirulent and virulent strain could be isolated from chickens regardless of the origin or vaccination status of the chickens. ND outbreaks among vaccinated flocks, suggesting that vaccination strategies have not effective yet in controlling the virus [36,37]. Antigenic similarity are shared among all NDV strains and these strains provides cross-protection against challenge with any other NDV strain. However, like most vaccines, NDV vaccines do not prevent vaccinated animals from becoming infected with NDV and subsequently shedding the virus [38]. Hidden immunosuppressive condition due micotoxicosis were also decreasing vaccination response [39]. Progressive genetic improvement on chicken phenotype might also increasing stress of the chicken resulting declined of immune response [40]. Matched between vaccines virus and circulating field isolates are important to provides better protection against transmission by reducing the magnitude of viral shedding. In the field, many factors play role on vaccine efficacy, thus making the antibody specificity become important. In endemic countries, aim of vaccination was not only on prevention of clinical disease and mortality, but also on decreasing the amount of virus shed from vaccinated birds [41].

Nevertheless, our findings indicates that NDV still possess threat to the poultry industries since this viruses maintained their evolution and circulation among vaccinated chickens. Some factors such stress, infection, and immunosuppressive might lead to progression of a full blown velogenic ND; If the disease progress to velogenic ND, it is going to be a potential threat to commercial poultry industry since the disease may go unnoticed and be left uncontrolled and at the end this might causing economic losses [36,37] of Indonesia’s poultry industry.

## Conclusions

RT-PCR and amino acid sequencing were developed for NDV detection and pathotyping. Our study showed that RT-PCR using F gene pathotype specific primers combination were able to differentiated virulent and avirulent NDV strain. Our result also provided evidence that virulent NDV strain was circulating among vaccinated flocks in West Java, Indonesia, which indicated vaccination program have not effective yet in controlling the virus, and this might possess a risk of causing ND outbreaks and causing economic losses within the poultry industry.

## Authors’ Contributions

DDP executed the work (collection of data, analysis, and writing of manuscript); EH participated in conception and design the study and drafting of the manuscript; AS participated in designed the study and drafting of the manuscript; RDS participated in designed the study, analysis of data and drafting of the manuscript, NLPIM participated in analysis and interpretation of data and drafting of the manuscript, ONP participated in analysis and interpretation of data and writing of manuscript; All authors read and approved the final manuscript.
